# Association of Serum Interleukin-8 and Serum Amyloid A With Anxiety Symptoms in Patients With Cerebral Small Vessel Disease

**DOI:** 10.3389/fneur.2022.938655

**Published:** 2022-07-08

**Authors:** Li-Li Shan, Yi-Lin Wang, Tian-Ci Qiao, Yue-Feng Bian, Ya-Jing Huo, Cen Guo, Qian-Yun Liu, Zi-Dong Yang, Ze-Zhi Li, Ming-Yuan Liu, Yan Han

**Affiliations:** ^1^Department of Neurology, Yueyang Hospital of Integrated Traditional Chinese and Western Medicine, Shanghai University of Traditional Chinese Medicine, Shanghai, China; ^2^Georgetown Preparatory School, North Bethesda, MD, United States; ^3^Institute of Science and Technology for Brain-Inspired Intelligence, Fudan University, Shanghai, China; ^4^Department of Psychiatry, The Affiliated Brain Hospital of Guangzhou Medical University, Guangzhou, China

**Keywords:** cerebral small vessel disease, anxiety symptoms, inflammatory factors, SAA, IL-8

## Abstract

**Objective:**

Cerebral small vessel disease (CSVD) is a clinical syndrome caused by pathological changes in small vessels. Anxiety is a common symptom of CSVD. Previous studies have reported the association between inflammatory factors and anxiety in other diseases, but this association in patients with CSVD remains uncovered. Our study aimed to investigate whether serum inflammatory factors correlated with anxiety in patients with CSVD.

**Methods:**

A total of 245 CSVD patients confirmed using brain magnetic resonance imaging (MRI) were recruited from December 2019 to December 2021. Hamilton Anxiety Rating Scale (HAMA) was used to assess the anxiety symptoms of CSVD patients. Patients with HAMA scores ≥7 were considered to have anxiety symptoms. The serum levels of interleukin-1β (IL-1β), IL-2R, IL-6, IL-8, IL-10, tumor necrosis factor-α (TNF-α), serum amyloid A (SAA), C-reactive protein (CRP), high-sensitivity C-reactive protein (hs-CRP) and erythrocyte sedimentation rate (ESR) were detected. We compared levels of inflammatory factors between the anxiety and non-anxiety groups. Logistic regression analyses examined the correlation between inflammatory factors and anxiety symptoms. We further performed a gender subgroup analysis to investigate whether this association differed by gender.

**Results:**

In the fully adjusted multivariate logistic regression analysis model, we found that lower levels of IL-8 were linked to a higher risk of anxiety symptoms. Moreover, higher levels of SAA were linked to a lower risk of anxiety symptoms. Our study identified sex-specific effects, and the correlation between IL-8 and anxiety symptoms remained significant among males, while the correlation between SAA and anxiety symptoms remained significant among females.

**Conclusions:**

In this study, we found a suggestive association between IL-8, SAA, and anxiety symptoms in CSVD participants. Furthermore, IL-8 and SAA may have a sex-specific relationship with anxiety symptoms.

## Introduction

Cerebral small vessel disease (CSVD) represents a cluster of disorders in which pathological alterations are found in small arteries, arterioles, venules, and capillaries (1). The alterations of parenchymal visible on brain imaging are considered clinical hallmarks of CSVD (2). CSVD may lead to cognitive decline, dementia, gait impairment, mood disturbance, and stroke through multiple mechanisms (1, 3–5). Importantly, mood disturbances are very commonly identified in patients with CSVD (6–8). There exists evidence that MRI markers of CSVD are consistently associated with a higher incidence of depression (9–11). The vascular depression hypothesis postulates that CSVD leads to mood disorders via damage to brain structures or neural connections involved in mood regulation (9, 12). However, most small vessel disease lesions in CSVD patients are thought to be silent in clinically defined early stages, or clinical symptoms are often highly inconsistent with brain imaging (13). Accordingly, researchers have begun to focus on other predictors relevant to CSVD. Inflammation is attracting more attention as a risk factor and classical pathological feature of CSVD (14, 15).

On the other hand, early studies have suggested that chronic inflammation may potentially trigger mood disorders (16–19). CSVD is often accompanied by anxiety symptoms, and such symptoms might also be correlated with inflammation, as often reported in the healthy population. The relationship between serum inflammatory factor levels and anxiety disorder has been much less studied than its relationship with depression. Recent studies have examined whether low-grade inflammation may be contributing to the connection between anxiety disorders and cardiovascular diseases (CVDs) (20). The mechanism behind the link between inflammation and anxiety may be that inflammatory markers influence metabolic pathways that affect functions of the neurotransmitters, ultimately affecting the neurocircuits that regulate anxiety (21, 22). In the context of anxiety disorders, the most studied inflammatory markers are CRP, interleukin (IL-6), and tumor necrosis factor**-α** (TNF**-α**) (21, 23, 24). However, a prominent role of other inflammation in psychopathology cannot be excluded (25, 26).

To the best of our knowledge, studies on the relationship between inflammatory markers and anxiety symptoms in CSVD patients are sparse, and it is further unknown whether the relationship would differ by gender.

In this study, we hypothesize that inflammatory markers may be one of the possible mechanisms of anxiety symptoms in CSVD patients. We aim to investigate which inflammatory factors or cytokines are the most predictive in explaining anxiety symptoms in CSVD. Further understanding of underlying anxiety mechanisms in CSVD may help identify potential new therapeutic targets.

## Materials and Methods

### Study Design and Participants

The ethics committee of Yueyang Hospital of Integrated Traditional Chinese and Western Medicine, affiliated with the Shanghai University of Traditional Chinese Medicine, approved the study (Approval number: 2020–060). All subjects provided informed written consent. Patients with CSVD diagnosed using brain magnetic resonance imaging (MRI) admitted to the neurology department were sequentially screened from December 2019 to December 2021.

Inclusion criteria: 1. aged between 18 and 80 years, 2. patients with CSVD diagnosed by MRI based on the standards for reporting vascular changes on neuroimaging (STRIVE) (2), including typical radiological findings of lacunes of presumed vascular origin, white matter hyperintensity (WMH), moderate to severe (number of BG-EPVS > 10) basal ganglia enlarged perivascular spaces (BG-EPVS) (27) and cerebral microbleeds (CMB).

Exclusion criteria: 1. presence of speech or hearing impairment, unable to complete the questionnaires due to communication difficulties, 2. presence of severe organ dysfunction (liver and kidney diseases), immune diseases, cancer, acute infections, allergies, 3. presence of severe mental disorders, uncontrolled somatic diseases, 4. presence of contraindications to MRI, 5. presence of hereditary and amyloidosis or WMH caused by other reasons, 6. presence of Parkinson's disease, Alzheimer's disease or any other neurodegenerative disease, 7. presence stenosis of intracranial and extracranial large arteries (stenosis >50%), 8. patients treated with antipsychotic drugs within 2 weeks of initial screening.

### Inflammatory Markers

Fasting venous blood was collected from each individual in the morning after fasting for 12 h using Vacutainer K2 EDTA Tube (6.0 mL, #367863; Becton Dickinson, Franklin Lakes, NJ, USA) and Vacutainer SST II Tube (5.0 mL, #367955; Becton Dickinson, Plymouth, UK). Serum levels of interleukin-1β (IL-1β), IL-2R, IL-6, IL-8, IL-10, and tumor necrosis factor-α (TNF-α) were detected by using Siemens kits in Immulite 1000 automatic chemiluminescence immunoassay analyzer (Siemens Healthcare Diagnostics GmbH, Berlin, Germany). C-reactive protein (CRP) was measured by scattering immunoturbidimetry using Mindray BC-6800 automated hematology analyzer (Mindray Bio-Medical Electronics Co., Ltd, Shenzhen, China). Serum amyloid A (SAA) was measured by scattering immunoturbidimetry using an Astep Plus protein analyzer (Goldsite Diagnostics Inc., Shenzhen, China). High-sensitivity C-reactive protein (hs-CRP) was detected by immunoturbidimetry using Beckerman AU5841 automatic biochemical analyzer (Beckman Coulter Inc., Brea, CA, USA). Erythrocyte sedimentation rate (ESR) was detected by capillary spectrophotometry using the TEST1 ESR analyzer (Alifax, Padova, Italy). All analyses were performed in the clinical laboratory department, located within the Hospital. The manufacturers provided the kits, and laboratory professionals performed all operations strictly according to the manufacturer's instructions.

### Data Collection and Definitions

We evaluated all participants' Hamilton Anxiety Rating Scale (HAMA) scores (28). Demographic characteristics included sex, age, education, marital status (single, married, divorced, widowed, and remarried), living arrangements (living alone or living with others), and medical history. Since lifestyle was associated with both anxiety and inflammation, we assessed smoking status (never, occasional, current, former), alcohol intake (never, occasional, current, former), salt intake (≥6 g/day), physical activity, and body mass index (BMI, kg/m^2^). Patients with HAMA scores≥ 7 were included in the anxiety group and otherwise included in the non-anxiety group.

### Statistical Analysis

Continuous variables conforming to normal distribution were presented as mean ± standard deviation (SD), and differences between groups were assessed using an independent *t*-test. Non-normally distributed continuous variables were described with median (quartile), and the Wilcoxon rank-sum test was applied for difference comparison. Frequencies and percentages expressed categorical data, and the Chi-squared test or Fisher's exact test was used for the intergroup comparison. Univariate and multivariate logistic regression analyses examined associations between serum inflammatory factors and anxiety symptoms. We applied restricted cubic spline (RCS) analysis to evaluate further the possible non-linear relationship between the inflammatory markers and anxiety symptoms. Three different models were tested to account for potential confounders: Model 1: unadjusted; Model 2: adjustment for age and sex; Model 3: adjustment for age, sex, passive smoking, and physical activity. In addition, we performed a gender subgroup analysis to examine the above association. Odds ratios (OR) with their 95% confidence interval (CI) were reported. Alpha was set at 0.05. Statistical analyses were performed using SAS 9.4 software (SAS Institute Inc., Cary, NC, USA).

## Results

### Demographic and Clinical Characteristics of Participants

Two hundred forty-five CSVD patients with an average age of 59 years old were included in this study, and 51% of them were female. The clinical characteristics of CSVD participants with or without anxiety symptoms are presented in Table 1. Among CSVD patients, individuals with anxiety symptoms were more physically active (*p* = 0.036), and the proportion of passive smokers was higher (*p* = 0.037) than participants without anxiety symptoms. There were no other statistically significant differences in demographic or clinical characteristics between the anxiety and non-anxiety groups.

**Table 1:** Comparison of demographic information in CSVD patients with and without anxiety symptoms.

**Variables**	**Total (*n* = 245)**	**Group**		**Z/t/χ^2^**	***p*-Value**
		**Non-anxiety (*n* = 97)**	**Anxiety (*n* = 148)**		
Female*, n* (%)	126 (51.43)	51 (52.58)	75 (50.68)	0.085	0.771
Age (years), Mean±SD	59.26 ± 11.39	59.68 ± 11.33	58.99 ± 11.47	0.47	0.642
Marital status*, n* (%)				-	0.563
Single	8 (3.27)	3 (3.09)	5 (3.38)		
Married	216 (88.16)	87 (89.69)	129 (87.16)		
Divorced	7 (2.86)	4 (4.12)	3 (2.03)		
Widowed	13 (5.31)	3 (3.09)	10 (6.76)		
Remarried	1 (0.41)	0 (0.00)	1 (0.68)		
Residence*, n* (%)				0.775	0.379
Living alone	16 (6.53)	8 (8.25)	8 (5.41)		
Living with others	229 (93.47)	89 (91.75)	140 (94.59)		
Education level (years), Mean ± SD	11.71 ± 3.70	11.68 ± 3.81	11.74 ± 3.64	−0.12	0.908
BMI, Mean ± SD	24.05 ± 4.24	24.51 ± 4.03	23.76 ± 4.35	1.36	0.175
Smoking*, n* (%)				2.240	0.524
Never	147 (60.00)	60 (61.86)	87 (58.78)		
Occasional	9 (3.67)	2 (2.06)	7 (4.73)		
Current	53 (21.63)	23 (23.71)	30 (20.27)		
Former	36 (14.69)	12 (12.37)	24 (16.22)		
Passive smoking*, n* (%)	49 (20.00)	13 (13.40)	36 (24.32)	4.369	**0.037**
Drinking*, n* (%)				4.480	0.214
Never	113 (46.12)	45 (46.39)	68 (45.95)		
Occasional	78 (31.84)	27 (27.84)	51 (34.46)		
Current	35 (14.29)	19 (19.59)	16 (10.81)		
Former	19 (7.76)	6 (6.19)	13 (8.78)		
Salt intake (≥6 g/day)*, n* (%)	52 (21.22)	20 (20.62)	32 (21.62)	0.035	0.851
Physical activity, M (Q_1_, Q_3_)	15.00 (0.00,20.00)	12.00 (0.00,20.00)	15.00 (0.00,20.00)	−2.095	**0.036**
Cerebrovascular disease*, n* (%)	66 (27.39)	25 (26.04)	41 (28.28)	0.145	0.703
Hypertension*, n* (%)	111 (45.31)	49 (50.52)	62 (41.89)	1.759	0.185
Heart disease*, n* (%)	16 (6.53)	4 (4.12)	12 (8.11)	1.524	0.217
Diabetes*, n* (%)	45 (18.37)	16 (16.49)	29 (19.59)	0.376	0.540
Hyperlipidaemia*, n* (%)	76 (31.40)	24 (24.74)	52 (35.86)	3.336	0.068
Migraine headaches*, n* (%)	64 (26.23)	23 (23.71)	41 (27.89)	0.528	0.468

*Statistically significant values are identified in boldface*.

### Comparison of Inflammatory Markers Levels

The comparison results of the serum inflammatory factors between the two groups are shown in Table 2. The serum levels of IL-8 (*p* = 0.003) and SAA (*p* = 0.01) in the anxiety group were significantly lower than those in the non-anxiety group, while other markers of inflammation, including TNF-α, IL-1β, IL-2R, IL-6, IL-10, hs-CRP, CRP, and ESR did not demonstrate significant differences between the two groups.

**Table 2:** Comparisons of inflammatory markers between the two groups.

**Variables**	**Total (*n* = 245)**	**Group**		**Z/t**	***p*-Value**
		**Non-anxiety (*n* = 97)**	**Anxiety (*n* = 148)**		
TNF-α (pg/ml), M (Q_1_, Q_3_)	7.35 (5.90,9.10)	7.55 (6.30,9.70)	7.30 (5.85,8.80)	1.272	0.203
CRP (mg/L), M (Q_1_, Q_3_)	0.88 (0.49,2.21)	0.90 (0.42,2.43)	0.88 (0.53,2.03)	−0.260	0.795
IL-1β (pg/ml), M (Q_1_, Q_3_)	5.00 (5.00,8.30)	5.00 (5.00,8.65)	5.00 (5.00,8.07)	0.061	0.951
IL-2R (u/ml), M (Q_1_, Q_3_)	358.00 (283.00,432.00)	371.00 (286.00,425.00)	349.50 (279.00,440.00)	0.115	0.908
IL-6 (pg/ml), M (Q_1_, Q_3_)	2.56 (2.00,3.59)	2.69 (2.00,4.23)	2.44 (2.00,3.22)	1.829	0.067
IL-8 (pg/ml), M (Q_1_, Q_3_)	58.15 (30.00,124.00)	84.30 (37.30,155.00)	48.35 (23.50,108.50)	2.922	**0.003**
IL-10 (pg/ml), Mean±SD	5.06 ± 0.57	5.04 ± 0.29	5.08 ± 0.69	−0.64	0.523
SAA (mg/L), M (Q_1_, Q_3_)	3.09 (2.50,8.48)	5.51 (2.50,8.87)	2.50 (2.50,7.55)	2.583	**0.010**
hs-CRP (mg/L), M (Q_1_, Q_3_)	0.82 (0.45,1.87)	0.88 (0.43,2.58)	0.78 (0.46,1.47)	0.949	0.343
ESR (mm/h), M (Q_1_, Q_3_)	11.00 (5.00,18.00)	9.00 (5.00,18.00)	12.50 (5.00,18.00)	−0.500	0.617

*TNF-α, tumor necrosis factor α; CRP, C-reactive protein; IL, interleukin; SAA, serum amyloid A; hs-CRP, high-sensitivity C-reactive protein; ESR, erythrocyte sedimentation rate; Statistically significant values are identified in boldface*.

### Association of Inflammatory Markers With Anxiety Symptoms

**Figure 1:**
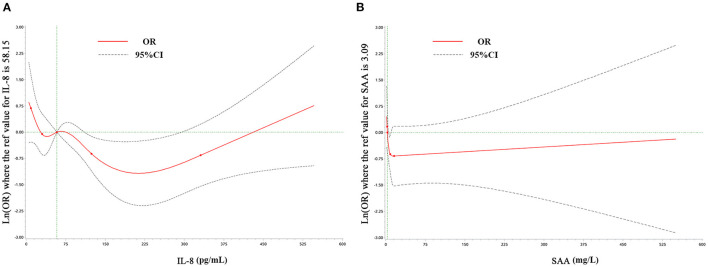
Examination of the relationship between IL-8 **(A)**, SAA **(B)**, and anxiety symptoms by restricted cubic splines analysis. Odds ratios are represented by the solid red line and the 95% confidence interval by the dotted line.

Restricted cubic spline (RCS) analysis revealed a non-linear relationship between IL-8, SAA, and anxiety symptoms, as shown in Figure 1. IL-8 and SAA were grouped in quartiles: first quartile (Q1; IL-8<30 pg/mL), Q2 (30 pg/mL≤ IL-8<58.15 pg/mL), Q3 (58.15 pg/mL≤ IL-8<124 pg/mL) and Q4 (IL-8≥124pg/mL), with the Q4 as the reference group; Q1(SAA <2.5 mg/L), Q2(2.5 mg/L≤SAA< 3.09 mg/L), Q3 (3.09 mg/L≤SAA<8.48 mg/L), Q4(SAA≥8.48 mg/L), with the Q2 as the reference group. Univariate and multivariate logistic regression were used to analyze the association between anxiety symptoms and inflammation markers and to adjust for possible confounding factors in CSVD patients, as shown in Table 3.

**Table 3:** Odds ratios (95%CI) of anxiety symptoms associated with IL-8 and SAA.

**Variables**	**Model1**		**Model2**		**Model3**	
	**OR (95%CI)**	***p*-Value**	**OR (95%CI)**	***p*-Value**	**OR (95%CI)**	***p*-Value**
**IL-8**						
Q_1_ (<30)	3.40 (1.56–7.41)	**0.002**	3.66 (1.65–8.14)	**0.001**	3.32 (1.47–7.53)	**0.004**
Q_2_ (30–58.15)	2.15 (1.02–4.49)	**0.043**	2.29 (1.08–4.86)	**0.031**	2.28 (1.05–4.91)	**0.036**
Q_3_ (58.15–124)	1.94 (0.93–4.05)	0.078	1.99 (0.95–4.18)	0.069	1.93 (0.90–4.10)	0.089
Q_4_ (≥124)	Ref		Ref		Ref	
**SAA**						
Q_1_(<2.5)	–		–		–	
Q_2_(2.5–3.09)	Ref		Ref		Ref	
Q_3_ (3.09–8.48)	0.64 (0.33–1.23)	0.175	0.63 (0.33–1.23)	0.176	0.62 (0.32–1.22)	0.164
Q_4_(≥8.48)	0.46 (0.24–0.88)	**0.020**	0.46 (0.24–0.88)	**0.019**	0.51 (0.26–0.98)	**0.045**

*Ref, Reference; Model 1: univariate logistic regression analysis; Model 2, adjusted for age and sex. Model 3, adjusted for age, sex, physical activity, and passive smoking; Statistically significant values are identified in boldface*.

Model 3 shows the associations after adjustment for age, sex, passive smoking, and physical activity. CSVD patients with lower levels of IL-8 were associated with a higher risk of anxiety symptoms. The odds ratios (OR) in the Q1 and Q2 were 3.32 (95% CI: 1.47–7.53, *p* = 0.004) and 2.28 (95% CI: 1.05–4.91, *p* = 0.036) compared with Q4, respectively. Patients with higher levels of SAA were associated with a lower risk of anxiety symptoms, the OR in Q4 was 0.51 (95%CI: 0.26–0.98, *p* = 0.045) compared with Q2.

### Subgroup Analysis in Gender

Significant gender interactions were found between anxiety symptoms and inflammation (29, 30), which led us to perform regression analyses within the subgroups of gender. The results are shown in Table 4. CSVD patients with lower IL-8 levels were associated with an increased risk of anxiety in males but not in females, and the adjusted OR with 95%CI were 3.94 (1.22–12.70, *p* = 0.022), 3.93 (1.22–12.62, *p* = 0.021) and 3.32 (1.14-9.61, *p* = 0.027) in Q1, Q2 and Q3 vs. Q4, respectively. Compared with the lower SAA levels in Q2, higher SAA levels in Q4 were associated with a decreased risk of anxiety symptoms in females (OR = 0.33, 95%CI:0.12–0.88, *p* < 0.028) but not males.

**Table 4:** Odds ratios (95%CI) of anxiety symptoms associated with IL−8 and SAA for gender subgroup analysis.

**Variables**	**Male**		**Female**	
	**OR (95%CI)**	***p*-Value**	**OR (95%CI)**	***p*-Value**
**IL−8**				
Q_1_ (<30)	3.94 (1.22–12.70)	**0.022**	2.53 (0.79–8.13)	0.119
Q_2_ (30–58.15)	3.93 (1.22–12.62)	**0.021**	1.32 (0.46–3.81)	0.609
Q_3_ (58.15–124)	3.32 (1.14–9.61)	**0.027**	1.12 (0.36–3.47)	0.848
Q_4_ (≥124)	Ref		Ref	
**SAA**				
Q_1_(<2.5)	–		–	
Q_2_(2.5–3.09)	Ref		Ref	
Q_3_ (3.09–8.48)	0.66 (0.23–1.88)	0.441	0.57 (0.23–1.42)	0.228
Q_4_(≥8.48)	0.64 (0.25–1.64)	0.351	0.33 (0.12–0.88)	**0.028**

*Ref, Reference; Statistically significant values are identified in boldface*.

## Discussion

It was previously shown that inflammation factors might contribute to mood disturbances in cardiovascular disease (CVD) patients (20, 31, 32). Nonetheless, the role of inflammation factors in anxiety symptoms identified in CSVD patients has not been described. Our study indicated that IL-8 or SAA levels in CSVD patients might be negatively correlated with the risk of anxiety symptoms. Furthermore, we found sex differences in the associations between anxiety symptoms and inflammation. However, potential biological mechanisms underlying the relationships between IL-8, SAA, and anxiety symptoms in CSVD patients remain unclear.

Research on serum levels of IL-8 in patients with mood disorders is limited and has yielded different results. Consistent with our findings, previous studies indicated that IL-8 levels were negatively correlated with anxiety symptom severity among suicide attempters displaying anxiety disorders (33–35) or antidepressant drug-naïve patients with major depressive disorder (MDD) (36). Under physiologic conditions, inflammatory cytokines play an important role in neuroplasticity and neurogenesis (37). Previous studies have revealed that IL-8 has neuroprotective and neurotrophic properties (38–40), such as IL-8 showed a protective effect on perinatal asphyxia brain injury (41). Mood disturbances in CSVD patients are associated with WMH, lacunar infarcts, and microbleeds (8). Our study indicated that decreased IL-8 levels were associated with an increased risk of anxiety symptoms. Therefore, we considered that IL-8 might play a neuroprotective effect in disrupting brain structures involved in anxiety regulation in CSVD patients.

Additionally, IL-8 can modulate neurotransmitter levels (42) and may be involved in the anxiety-related neuronal circuits, such as attenuating the serotonin and dopamine systems (33, 43). Therefore, we speculate that IL-8 may also involve the biological mechanisms regulating anxiety recovery in CSVD. Indeed, physiologic levels of inflammatory factors are necessary for many neurophysiological processes associated with the protection of mood disturbance (44). When IL-8 levels are too low, the physiological processes of homeostasis may be disrupted.

Accumulating evidence suggests that SAA has proinflammatory properties (45–47). Nevertheless, some of the recent findings on SAA suggested that the primary role of SAA may be associated with homeostasis rather than proinflammatory (48). For example, studies have shown that SAA promotes the resolution of inflammation and tissue repair and regulates the homeostasis of the inflammatory process by inducing M2-like macrophages (49). Furthermore, several studies have found that systemic administration of SAA could not increase the production of proinflammatory cytokines (50, 51). The potential explanations for consideration might be that masses of reports on the proinflammatory effects of SAA were using recombinant human SAA (rhSAA) (45, 52, 53). Nevertheless, natural SAA from serum or plasma lacks most of the proinflammatory activity shown by rhSAA (51, 54, 55).

There are few direct clinical studies relating SAA to vascular diseases, and its essential biological role remains poorly understood. The levels of SAA in the blood of healthy individuals are generally below 3 mg/L (56). However, their levels can transiently spike 1000-fold 24 h after the onset of the acute-phase response (57) and then return to a low circulating baseline once the event resolves. The various functions ascribed to SAA are dose-dependent. Their role in inflammation can vary depending on the amount of cytokine expressed and the length of expression or the form of receptor activated by cytokine (55). A stable level of SAA is essential for most biological systems. Small increases in SAA levels may also be associated with the diagnosis or prognosis of a specific disease.

In our study, SAA levels did not show a 1000-fold increase, indicating low levels of peripheral inflammatory activity in the early stage of the CSVD. Compared with the lowest serum SAA levels in Q2 (2.5–3.09 mg/L), the Q4 (≥8.48 mg/L) group was associated with a decreased risk of anxiety symptoms (OR = 0.51, 95%CI: 0.26–0.98, *p* = 0.045), suggesting that significant but smaller increases of SAA levels may protect the patients against the early course of inflammation in CSVD. Additionally, previous studies have found that relatively low concentrations of SAA induce neutrophils to release IL-8 in a dose-dependent manner (46, 58). Thus, it could be hypothesized that IL-8 and SAA may both implicate the underlying pathophysiology of anxiety symptoms in the early stages of CSVD.

There is evidence that inflammatory profiles in mood disorders differ between females and males (30, 59–61). Concerning gender differences, our study found that anxiety symptoms were associated with IL-8 levels (males only) and SAA levels (females only) in CSVD patients. It may be that estradiol increases the secretion of IL-8 in immature dendritic cells, while androgen is generally immunosuppressive (62). Similarly, previous observational studies found higher IL-8 levels associated with decreased depressive symptoms in males (29). In contrast to our findings, Kruse et al. have reported that IL-8 levels are negatively correlated with total HAMD score among females but not males in cross-sectional studies of depressed patients (63). These similar sex-specific effects were also found in electroconvulsive or ketamine therapy studies in depressed patients (64, 65). However, not all studies have been consistent, reflecting the between-study heterogeneity within the diagnostic categories of depression and anxiety or distinct study designs.

## Conclusion

To sum up, various serum inflammatory factors and mechanisms are in place to maintain homeostasis, but dysregulation of their actions often contributes to diseases, including anxiety symptoms in CSVD patients. The underlying pathophysiology of IL-8 and SAA in CSVD with anxiety symptoms is complex and needs further investigation.

## Limitations

This study is a single-center cross-sectional study based on a relatively small sample. We cannot clarify the causal relationship. A large cohort corroboration is required to explore whether inflammation is a precursor, consequence, or bidirectional relationship in CSVD patients with anxiety symptoms. Additionally, we examined circulating levels of inflammatory factors but did not assess inflammatory factor levels in the cerebrospinal fluid. Further clinical trials are needed to guide the detection of inflammatory factors in CSVD patients with anxiety symptoms.

## Data Availability Statement

The raw data supporting the conclusions of this article will be made available by the authors, without undue reservation.

## Ethics Statement

The studies involving human participants were reviewed and approved by Ethics Committee of Yueyang Hospital of Integrated Traditional Chinese and Western Medicine, Shanghai University of Traditional Chinese Medicine (Approval ID: 2020-060). The patients/participants provided their written informed consent to participate in this study.

## Author Contributions

YH and M-YL designed the study. T-CQ and Y-FB collected data. L-LS and Y-LW analyzed the data and drafted this manuscript. Q-YL, Y-JH, CG, Z-DY, and Z-ZL were involved in the design and revision of the manuscript. All authors contributed to this article and approved the submitted version.

## Funding

This study was supported, in part, by grants from the National Key Research and Development Program of China (No. 2019YFC1711603), the Major Clinical Study Projects of Shanghai Shenkang Hospital Development Center (No. SHDC2020CR2046B), and Shanghai Science and Technology Development Foundation (No. 19401972802).

## Conflict of Interest

The authors declare that the research was conducted in the absence of any commercial or financial relationships that could be construed as a potential conflict of interest. The reviewer DW declared a shared parent affiliation with the author Z–DY to the handling editor at the time of review.

## Publisher's Note

All claims expressed in this article are solely those of the authors and do not necessarily represent those of their affiliated organizations, or those of the publisher, the editors and the reviewers. Any product that may be evaluated in this article, or claim that may be made by its manufacturer, is not guaranteed or endorsed by the publisher.
